# Proteomic study of different culture medium serum volume fractions on RANKL-dependent RAW264.7 cells differentiating into osteoclasts

**DOI:** 10.1186/s12953-015-0073-6

**Published:** 2015-05-02

**Authors:** Qi Xiong, Lihai Zhang, Lingli Xin, Yanpan Gao, Ye Peng, Peifu Tang, Wei Ge

**Affiliations:** Department of Orthopedics, General Hospital of Chinese PLA, Fuxing Road 28# Haidian district, Beijing, 100853 China; National Key Laboratory of Medical Molecular Biology & Department of Immunology, Institute of Basic Medical Sciences, Chinese Academy of Medical Sciences, DongdanSantiao 5# Dongcheng district, Beijing, 100005 China; Department of Obstetrics and Gynecology, The Second Artillery General Hospital of Chinese PLA, Xinjiekouwai Street 16# Xicheng district, Beijing, 100088 China

**Keywords:** Osteoclast, Cell Culture, Proteomics, Bioinformatics

## Abstract

**Background:**

Cultivation of osteoclasts is a basic tool for investigating osteolytic bone diseases. Fetal bovine serum (FBS) is the standard supplement used for *in vitro* cell culture medium. Typically, the serum volume fraction used for osteoclast cultivation is 10%. In this study, we investigated the use of a low serum (1% FBS) model for culturing osteoclasts.

**Results:**

To confirm the validity of this model for use in osteoclast research, we compared the capacity for osteoclastogenesis and bone resorption of RANKL-induced RAW 264.7 cells cultured in medium supplemented with 10% FBS and 1% FBS. Osteoclasts were successfully generated in medium supplemented with 1% FBS, and exhibited prolonged longevity and similar bone resorbing ability to those generated in medium supplemented with 10% FBS, although the osteoclasts were smaller in size. Proteomics and bioinformatics analyses were performed to assess the suitability of osteoclasts formed in low serum-containing medium for use in research focusing on osteoclast differentiation and function. Our study demonstrated that a total of 100 proteins were differentially expressed in cells cultured in medium containing 1% FBS, of which 29 proteins were upregulated, and 71 proteins were downregulated. Bioinformatics analysis showed that the electron transport chain and oxidative phosphorylation pathways were downregulated obviously; however, the osteoclast signaling pathway was unaffected. The data have been deposited to the ProteomeXchange with identifier PXD001935.

**Conclusion:**

Our study provides clear evidence of the validity of the low serum model for use in studying RANKL-dependent osteoclasts differentiation and bone resorption with the advantage of prolonged survival time.

**Electronic supplementary material:**

The online version of this article (doi:10.1186/s12953-015-0073-6) contains supplementary material, which is available to authorized users.

## Background

Osteoclasts are bone resorbing cells that are formed by the fusion of monocyte/macrophage cells induced by receptor activator of nuclear factor B ligand (RANKL), which is a member of the tumor necrosis factor superfamily [1,2]. Arthritis, osteoporosis, bone metastasis and other bone degrading diseases partially result from the abnormal function or overactivation of osteoclasts [3-5]. Given the critical role that osteoclasts play in such bone diseases, many studies of osteoclasts have been conducted. The cultivation of cells *in vitro* is an indispensable tool in basic cell and molecular biology studies [6] and culturing osteoclasts *in vitro* is an essential basis for exploring bone metabolism and the mechanisms of bone diseases.

Due to the identification of the RANKL/RANK signaling pathway as a crucial requirement for osteoclast formation, mature osteoclasts can now be obtained *in vitro*. Previously, *in vitro* models of osteoclast differentiation were mainly based on primary cell cultures, such as bone marrow macrophages, splenocytes, and peripheral blood monocytes induced by M-CSF and RANKL, all of which are poorly suited to molecular studies because of their limited availability and failure to produce pure populations of osteoclasts [7,8]. More recently, the mouse macrophage cell line RAW 264.7, a RANK-expressing cell line, is increasingly being used as a cellular model of osteoclast formation and function [2,9]. To obtain mature osteoclasts, RAW 264.7 cells are cultured in medium supplemented with FBS, and stimulated with RANKL. Conventionally, the serum volume fraction used is 10%; however, the use of varying serum concentrations in the osteoclast culture system has been reported. In fact, Vincent *et al*. demonstrated that RAW 264.7 cells cultured in serum-deprived medium could differentiate into tartrate resistant acid phosphatase (TRAP)-positive multinucleated osteoclasts, and their resorptive activity was significantly enhanced [10]. Nevertheless, the molecular changes associated with the differentiation were not mentioned in this study. Thus, the effects of the culture medium serum volume fraction on osteoclast formation and bone resorbing ability are still poorly defined.

To address these issues, we evaluated RANKL-dependent osteoclast formation (unless otherwise specified, osteoclast formation refers to RANKL-dependent osteoclast formation in present study) in both the conventional (10% FBS) and low serum (1% FBS) culture systems. The bone resorptive activities of the osteoclasts were investigated and the protein changes of RAW 264.7 cells cultured under both sets of conditions were compared in proteomics analyses, which provide a global view of protein expression changes in cells. Herein, we determined that the low serum fraction model did not interfere with the osteoclast signaling pathways and both culture systems could be used to obtain osteoclasts. Thus, either culture system can be used for specific applications according to the duration and intervention factors of the experiments.

## Results

### Osteoclastogenesis in medium supplemented with 10% FBS and 1% FBS

**Figure 1:**
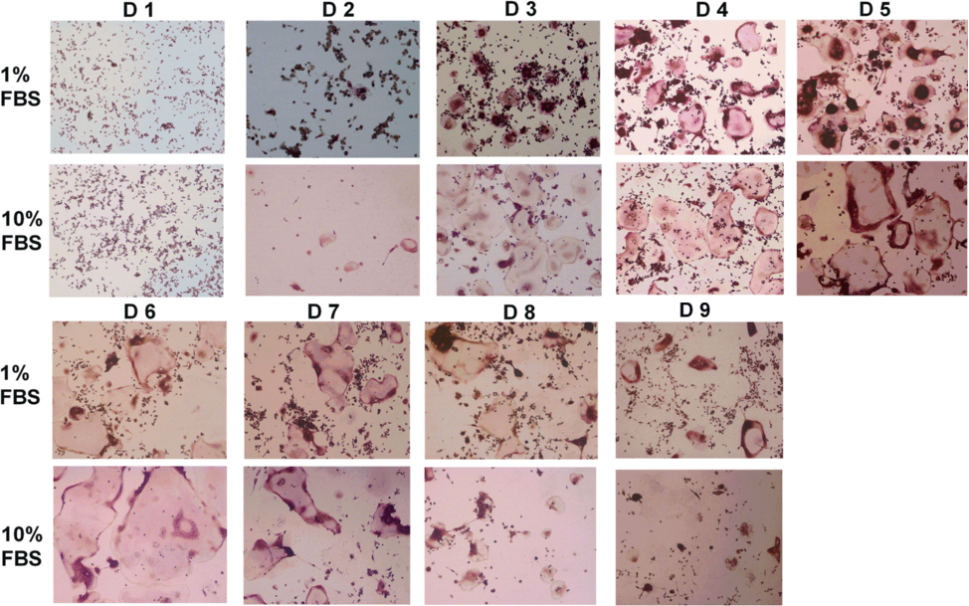
TRAP staining of RANKL-stimulated RAW264.7 cells in medium supplemented with 1% FBS and 10% FBS for 9 consecutive days. Multinucleated osteoclasts were apparently observed on Day 3 in both culture conditions. Osteoclasts formed in medium supplemented with 10% FBS were larger in size than those formed in medium supplemented with 1% FBS. Osteoclast debris was observed from Day 4 in medium supplemented with 10% FBS, but not until Day 6 in the cultures using medium supplemented with 1% FBS.

**Figure 2:**
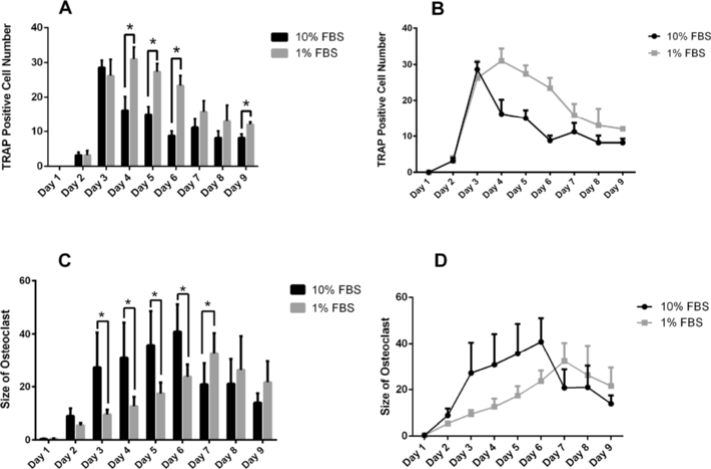
Comparison of TRAP-positive cell numbers and size of osteoclasts formed in medium supplemented with 10% FBS and 1% FBS. **(A)**: significantly more osteoclasts were observed in 1% FBS medium on Days 4, 5, 6 and 9; **(B)**: the number of osteoclasts reached a maximum in 10% FBS medium on Day 3, after which the numbers declined dramatically, while the number of osteoclasts reached a maximum in 1% FBS medium on Day 4 and subsequently decreased moderately. **(C)**: Osteoclasts formed in 10% FBS medium were obviously larger on Days 3, 4, 5 and 6, while osteoclasts formed in 1% FBS medium were significantly larger on Day 7. **(D)**: Osteoclasts formed in 10% FBS medium reached maximum size on Day 6, while osteoclasts formed in 1% FBS medium reached maximum size on Day 7. *: *P* < 0.05.

To compare osteoclastogenesis under both sets of conditions, RANKL-induced RAW 264.7 cells were cultured in medium supplemented with 10% FBS or 1% FBS. TRAP staining was performed to confirm the formation of mature multinucleated osteoclasts. Osteoclast formation was observed in both media from Day 2. In medium supplemented with 10% FBS, the number of osteoclasts reached a peak on Day 3, with a subsequent rapid decline. In contrast, the number of osteoclasts achieved a maximum on Day 4 in the medium supplemented with 1% FBS, with a subsequent marginal decline. Statistical analysis demonstrated that the number of multinucleated osteoclasts formed in medium supplemented with 10% FBS was slightly greater than that in medium supplemented with 1% FBS during the first three days. Subsequently, more multinucleated osteoclasts were generated in the medium supplemented with 1% FBS compared with that containing 10% FBS. There were significantly more osteoclasts in the low serum fraction medium on Days 4, 5, 6 and Day 9 (*P* < 0.001). Moreover, osteoclast debris was observed from Day 4 in medium supplemented with 10% FBS, but not until Day 6 in the cultures using medium supplemented with 1% FBS. Taken together, these observations showed that greater numbers of osteoclasts were formed in medium supplemented with 1% FBS, and that these osteoclasts exhibited prolonged survival compared with those formed in medium supplemented with 10% FBS (Figures 1and 2).

### Comparison of size of osteoclasts and area of resorbing surface

**Figure 3:**
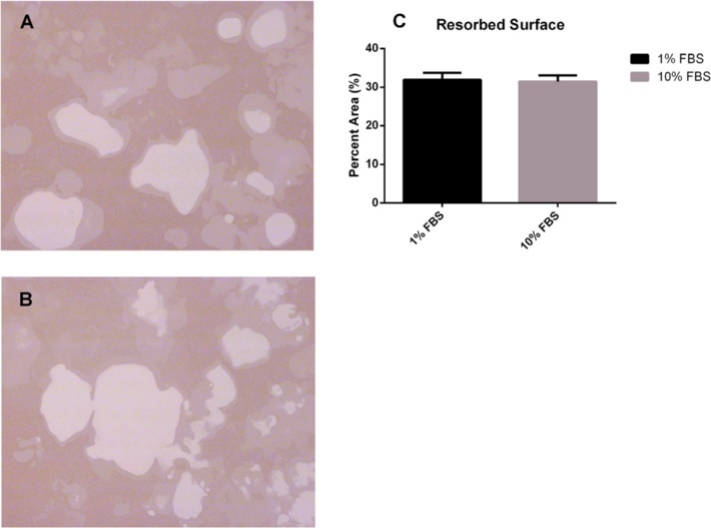
Bone resorptive capacity of osteoclasts was compared on Day 9. Toluidine blue staining was performed. **(A)**: resorptive pits formed by osteoclasts cultured in 1% FBS medium. **(B)**: resorptive pits formed by osteoclasts cultured in medium with 10% FBS. **(C)**: no difference in the area of resorptive pits was observed between the two culture conditions.

In addition to the comparison of numbers, osteoclast function was also compared. To compare the bone resorption capacity of osteoclasts formed in both culture systems, the size of osteoclasts and the area of the resorbing surface were evaluated. Osteoclasts formed in medium containing 10% FBS had increased in size dramatically by Day 3, enlarging to a maximum size by Day 6, followed by a reduction in size. In contrast, the size of osteoclasts formed in medium containing 1% FBS increased continuously to Day 7, and subsequently declined in size. Our study showed that the size of osteoclasts formed in medium supplemented with 10% FBS were significantly larger than those formed in medium supplemented with 1% FBS from Day 3 to Day 6 (*P*-values, 0.018, 0.018, 0.016 and 0.008, respectively). Unexpectedly, osteoclasts formed in medium containing 1% FBS were larger in size than those formed in medium supplemented with 10% FBS during the last three days of culture, especially on Day 7 (*P* = 0.049). In contrast to the cell size, the areas of the resorbing surfaces were similar under both sets of culture conditions (*P* = 0.753). In general, the mean size of osteoclasts was larger in the medium supplemented with 10% FBS from Day 2 to Day 6, while osteoclasts were larger during the last 3 days of culture in medium containing 1% FBS. However, there were no obvious differences in bone resorptive capacity between the osteoclasts formed under both sets of culture conditions (Figures 2 and 3).

### Identification and classification of differentially expressed proteins

**Figure 4:**
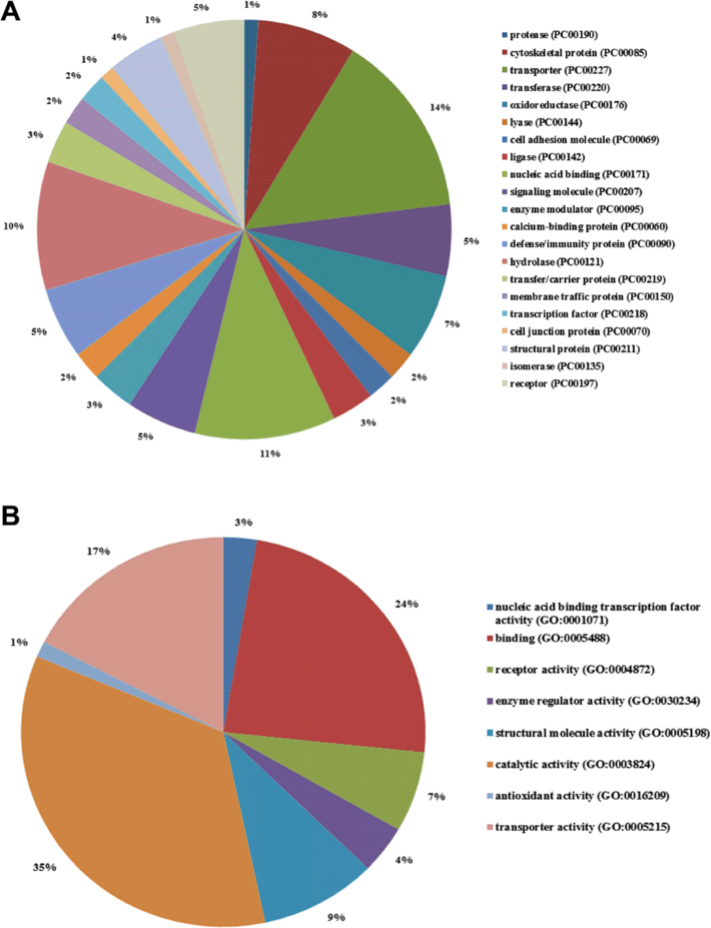
Classification of differentially expressed proteins using the PANTHER Classification System online. **(A)**: 21 categories of protein class hits identified in the PANTHER Protein Class Database. **(B)**: eight categories of molecular function hits identified in the GO Molecular Function database.

Proteomics analysis was conducted to evaluate the differential expression of proteins in RAW 264.7 cells cultured in medium supplemented with 10% FBS or 1% FBS. A total of 6,403 RAW264.7 cell proteins were identified in our study. A total of 100 differentially expressed proteins were identified by comparing the cells cultured under the two sets of conditions. Of these, 29 proteins were upregulated and 71 were downregulated in cells cultivated in medium with 1% FBS (Additional file 1). The PANTHER online analysis toolkit was used to classify the differentially expressed proteins. Eighty-five of 100 altered proteins were identified as hits in 21 categories of protein class in the PANTHER online database. The three categories with the greatest number of differentially expressed proteins were transporter (PC00227), nucleic acid binding (PC00176), and hydrolase (PC00121). Furthermore, we analyzed the molecular functional processes in which the differentially expressed proteins were involved. Of 100 altered proteins, 70 hits in eight categories of molecular function were identified in the PANTHER online database, with the majority of the proteins participating in catalytic activity (GO:003824), binding (GO:000548), and transporter activity (GO:0005215) (Figure 4).

### Bioinformatics analysis of differentially expressed proteins

**Figure 5:**
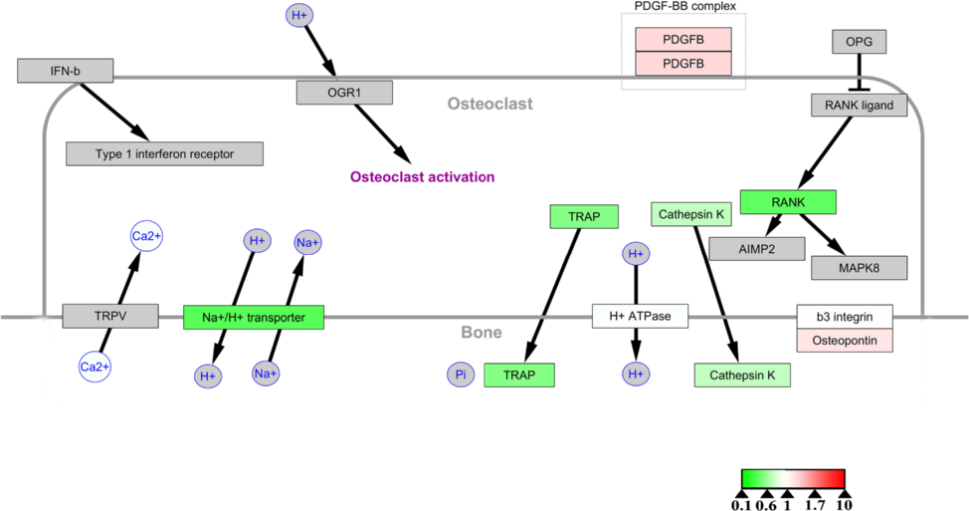
Visualization of all the proteins in the osteoclast signaling pathways identified in the WikiPathways database. Gray boxes indicate proteins not identified in our study; green boxes indicate downregulated proteins; red boxes indicate upregulated proteins. Color intensity is adjusted to indicate the ratio value.

**Figure 6:**
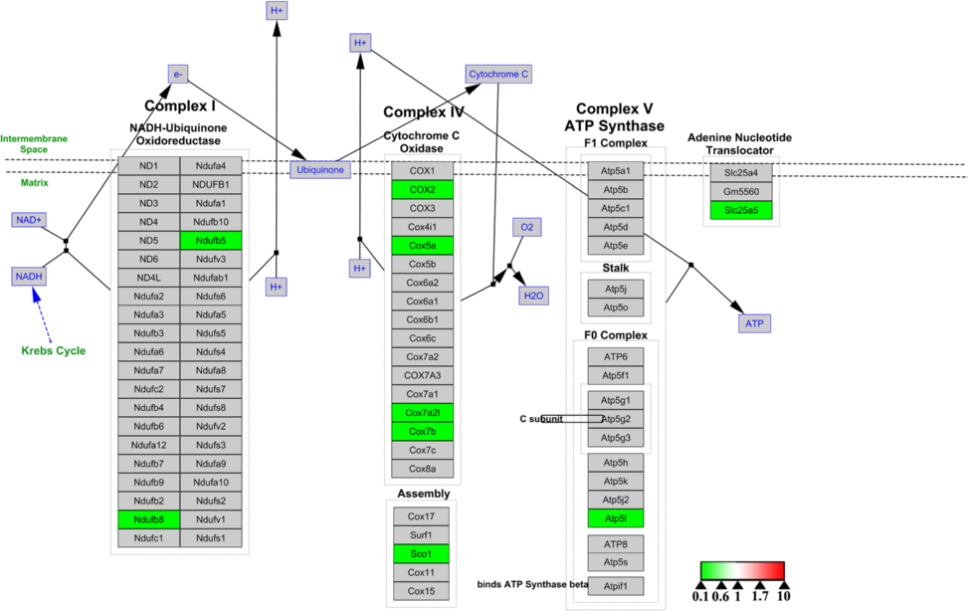
Visualization of differentially expressed proteins in the ETC. pathway pathways identified in the WikiPathways database. Gray boxes indicate proteins not identified as differentially expressed proteins; green boxes indicate downregulated proteins. Color intensity is adjusted to indicate the ratio value.

To investigate the molecular mechanisms underlying the differences between the osteoclasts formed under the two sets of conditions, we conducted bioinformatics analysis and visualization of the altered proteins with WebGestalt online tools and Cytoscape software. Only the electron transport chain pathway, in which only nine of the 100 altered proteins were involved, was identified in enrichment analysis based on the WikiPathways database. In addition, KEGG enrichment analysis highlighted six pathways, of which the metabolic pathway and oxidative phosphorylation involved 13 and 6 of 100 altered proteins, respectively, while three of the pathways were related to neurological diseases and one was associated with cardiac muscle contraction (Table 1). Cytoscape software was then used to visualize all the osteoclast signaling proteins identified in the WikiPathways database. Osteoclast biomarkers such as TRAP, RANK, cathepsin K were all slightly decreased (Figure 5), while osteopontin (OPN), a potent stimulator of osteoclastogenesis and resorptive activity [11], was slightly upregulated, suggesting that osteoclast bone resorptive activity was compromised to an extent. Cytoscape software was then used to visualize pathways involving the differentially expressed proteins. In the electron transport chain pathway, all nine proteins identified as hits in the WikiPathways database were downregulated, indicating inhibition of that pathway of energy production (Figure 6).

**Table 1:** **Enriched KEGG pathways analysis**

**Pathway name**	**Gene**	**EntrezGene**	**Statistics**
Huntington's disease	9	12858 11740 22335 66142 67264 14775 17709 22333 20463	C = 197;O = 9;E = 0.27;R = 33.89;rawP = 7.62e-12;adjP = 1.37e-10
Parkinson's disease	8	12858 11740 22335 66142 67264 17709 22333 20463	C = 148;O = 8;E = 0.20;R = 40.10;rawP = 3.05e-11;adjP = 2.75e-10
Metabolic pathways	13	12858 56012 *18263* ^*#*^ *14085* ^*#*^ *18770* ^*#*^ 66142 13200 *208715* ^*#*^ 67264 27425 *20018* ^*#*^ *110196* ^*#*^ 17709	C = 1184;O = 13;E = 1.60;R = 8.14;rawP = 6.66e-09;adjP = 4.00e-08
Oxidative phosphorylation	6	67264 12858 27425 17709 66142 20463	C = 147;O = 6;E = 0.20;R = 30.28;rawP = 5.36e-08;adjP = 2.41e-07
Cardiac muscle contraction	4	12858 17709 66142 20463	C = 81;O = 4;E = 0.11;R = 36.63;rawP = 4.70e-06;adjP = 1.69e-05
Alzheimer's disease	5	67264 12858 17709 66142 20463	C = 188;O = 5;E = 0.25;R = 19.73;rawP = 5.98e-06;adjP = 1.79e-05

•C: the number of reference genes in the category.

•O: the number of genes in the gene set and also in the category.

•E: the expected number in the category.

•R: ratio of enrichment.

•rawP: *P*-value from hypergeometric test.

•adjP: *P*-value adjusted by the multiple test adjustment.

•#: the genes upregulated.

## Discussion

Fetal bovine serum (FBS) is a cocktail of numerous factors required for cell maintenance, and is utilized routinely to supplement medium for *in vitro* cultivation of numerous cell-types, including osteoclasts [12]. Culture media are typically supplemented with 10% FBS [12,13]; however, this parameter does not generally mimic the in vivo microenvironment.

In the present study, osteoclast formation was investigated in medium supplemented with 1% FBS, which simulates physiological conditions more closely than 10% FBS. Here, we demonstrate the successful formation of osteoclasts with similar bone resorbing ability from RANKL-induced RAW264.7 cells cultured in media supplemented with either 10% FBS or 1% FBS. However, larger osteoclasts were formed more rapidly in medium supplemented with 10% FBS compared with those formed using the low serum model, while the longevity of the osteoclasts was less prolonged. Subsequent proteomics analysis of the molecular mechanisms underlying these differences revealed a total of 100 differentially expressed proteins involved in 12 biological processes. Of these, 29 proteins were upregulated and 71 were downregulated. However, no significant changes in the expression of proteins involved in osteoclastogenesis pathways were detected.

Osteoclasts are formed in the monocyte/macrophage lineage from hematopoietic progenitors. Osteoclastogenesis includes a number of steps comprised of survival, differentiation, fusion and activation [14]. Extensive investigations have demonstrated that the RANKL-mediated signaling pathway and downstream transcription factors play essential roles in the regulation of osteoclastogenesis. RANKL performs crucial regulation of osteoclastogenesis mediated by binding to its receptor, RANK, leading to the expression of a variety of osteoclast genes including TRAP, cathepsin K, calcitonin receptor, αvβ3-integrin and MMP-9 [15,16]. During osteoclastogenesis, RANKL induces the recruitment of TNF receptor-associated cytoplasmic factor 6 (TRAF6), which subsequently stimulates downstream signaling pathways, including IκB kinase (IKK), nuclear factor κB (NF-κB), c-Jun N-terminal kinase (JNK), Akt, c-Src, p38, ERK, activator protein 1 (AP-1), and nuclear factor and activator of transcription (NFATc1) [17,18]. No significant changes in the expression of these proteins were detected in this study, and the bone resorption capacity of osteoclasts formed under the two sets of conditions was similar. Hence, our results indicate that low serum medium does not change the expression of osteoclast biomarkers. In accordance with the observations reported by Vincent *et al*. [10], the osteoclasts formed in low serum medium in our study were smaller than those formed in medium supplemented with 10% FBS. Taken together, these observation indicate that osteoclasts formed in both mediums are similar, and confirm the validity of their use in research focusing on osteoclast differentiation and function.

Notably, our results revealed a significant difference in the size of osteoclasts generated in the two types of media. Therefore, we performed proteomics analysis and bioinformatics analysis to elucidate the molecular mechanisms underlying this difference. Our results clearly demonstrate downregulation of the electron transport chain (ETC.) and oxidative phosphorylation pathways, both of which are essential for ATP synthesis, in osteoclasts formed under low serum conditions. Previous studies have indicated that osteoclast formation is an energy consuming processes [19,20]. An *et al*. suggested that metabolism and re-direction of energy flow plays a critical role in osteoclast formation [21]. Therefore, we imply that energy restriction partially contributed to the small size of osteoclasts formed in medium supplemented with 1% FBS. Additionally, studies have shown that perturbations in some mitochondrial ETC. complexes increase lifespan [22-24]. Thus, it can be speculated that these reports explain our observation that osteoclasts formed in medium containing 1% FBS exhibited prolonged long-term survival, which might be beneficial for transgene studies of osteoclasts.

Previous studies have shown that increased mitochondrial ETC. activity enhances bone resorption by osteoclasts [25]; however, in our study, no differences in the bone resorption ability of osteoclasts formed under both sets of conditions were observed despite the downregulation in ETC. activity observed under low serum conditions. This may be due to the hypoxic conditions under which osteoclasts were cultured in the previous study. Knowles *et al*. reported that osteoclasts exposed to a constant hypoxic environment exhibited increased bone resorption ability in a HIF-1α-dependent manner [26]. Furthermore, Muzylak *et al*. found that osteoclasts generated in hypoxia showed an eight-fold increase in size compared with those cultured in normoxia [27]. Moreover, bone resorption requires osteoclast attachment to bone surfaces and the formation of the ruffled border by αvβ3 and small Rho family GTPases [28]. H^+^ ions are then pumped through the ruffled border to demineralize inorganic materials, and cathepsin K is secreted to digest organic materials [29]. No significant differences in the expression of these proteins were detected in the current study, indicating that low serum culture conditions do not affect the bone resorption capacity of osteoclasts. In addition, we found that OPN was slightly upregulated, which might compromise ETC. activity. It is worth noting that researchers studying osteoclast energy metabolism should avoid culturing osteoclasts in low serum medium due to the interference in the metabolic and ETC. pathways.

## Conclusions

In summary, compared with the typical culture conditions of medium supplemented with 10% FBS, cultivation of under low serum conditions (1% FBS) is associated with changes in a number of biological pathways in RAW 264.7 cells and influences osteoclasts formation without affecting bone resorption ability. Moreover, osteoclasts formed in medium supplemented with 1% FBS exhibited extended longevity. Accordingly, low serum conditions are suitable for studies requiring prolonged osteoclast survival, while these conditions should be avoided for studies of osteoclast metabolism. Our study provides an alternative model for studying osteoclast differentiation and bone resorption.

## Materials and methods

### Cells and reagents

RAW 264.7 cells were obtained from the Chinese Academy of Medical Sciences (Beijing, China). α-MEM (11095–080) and FBS (26140079) were obtained from Life Technologies. Recombinant mouse RANKL (462-TEC-010) was purchased from R&D Systems. Acid Phosphatase Leukocyte Kits (387–1) were purchases from Sigma Aldrich. Urea (17-1319-01) was obtained from GE healthcare. Protease inhibitor cocktails were obtained from Roche. TMT sixplex Isobaric Label Reagent Set (90061) was purchased from Thermo Scientific. Trypsin/Lys-C Mix (V5072) was purchased from Promega. BCA Protein Assay Kits (23227) were obtained from Thermo Scientific. Osteo Assay Surface 24 Well Plate (3987) was purchased from Corning.

### RAW264.7 cell cultivation and osteoclastogenesis

RAW 264.7 cells at a density of 1.5 × 10^5^ cells/ml in 6-well plates were cultured in α-MEM supplemented with either 10% (v/v) or 1% (v/v) FBS at 37°C in a 5% CO_2_ incubator; both groups were prepared in triplicate. On the next day, RAW 264.7 cells were harvested and used for subsequent proteomics analysis; the triplicates of each group were pooled and prepared for TMT labeling. To generate mature multinucleated osteoclasts, 30 ng/ml RANKL was added to both culture systems in 24-well plates. Cells were cultured for 9 days; both conditions were prepared in triplicate. The culture medium was refreshed every other day.

### TRAP staining

TRAP staining was performed to confirm the formation of mature osteoclasts. Mature osteoclasts were defined as TRAP-positive cells containing three of more nuclei. TRAP staining was performed using the Acid Phosphatase Leukocyte kit according to the manufacturer’s instructions. The number of TRAP-positive multinucleated osteoclasts was counted using inverted microscopy. Furthermore, the mean size of TRAP-positive multinucleated osteoclasts was calculated with Adobe Photoshop CS3 software in three random visual fields for each group.

### Osteoclast resorption pit assay

An Osteo Assay Surface 24 Well Plate was used to evaluate the bone resorptive activities of osteoclasts. On Day 9, the medium from the wells was aspirated and 100 μl of 10% bleach solution was added to each well and incubated for 5 minutes. Toluidine blue staining was then performed to improve the contrast for resorbed pit image analysis. NIH Image J software was used to assess the total area of the resorbed pits.

### Sample preparation and TMT labeling

Sample preparation and labeling were performed as described previously [30]. In brief, cells were washed with cold PBS three times followed by the addition of lysis buffer. Cell lysates were clarified by centrifugation at 16,000 g for 10 minutes at 4°C. Supernatants were obtained and protein concentrations were measured with the BCA Protein Assay Kit. Then, 100 μg of protein was incubated in 10 mM dithiothreitol at 50°C for 1 h. Thereafter, proteins were incubated with 25 mM indole acetic acid in the dark for 2 h. Proteins were then digested with trypsin/ Lys-C Mix at a protein/protease ratio of 25:1 and incubated overnight at 37°C. The TMT Isobaric Label Reagent Set was used to labeling proteins following the manufacturer’s manual protocol. The proteins extracted from cells cultured in medium with 10% FBS or 1% FBS were labeled with 0.8 mg TMT6-126 or TMT6-127, respectively. Equal amounts of the labeled protein digests from each group were combined for mass spectrometry (MS) analysis.

### High-performance liquid chromatography (HPLC)

Fractionation of combined protein digests was conducted as described previously [31]. Briefly, the combined TMT labeled samples were dissolved in 100 μl 0.1% FA and transferred to a MS tube for HPLC analysis (UltiMate 3000 UHPLC, Thermo Scientific) using an Xbridge BEH300 C18 column (4.6 × 250 mm^2^, 5 μm, 300 Å, Waters). Fifty fractions were collected into microtubes at 1.5 min intervals. The fractions were dried under vacuum and dissolved in 20 μl 0.1% FA for further LC-MS/MS analysis.

### LC-MS/MS analysis

LC-MS/MS was performed using a Q Exactive mass spectrometer. Following separation using a 120 min gradient elution at a flow rate of 0.3 μl/min using UltiMate 3000 RSLCano System (Thermo Scientific), the protein digests were analyzed with a directly interfaced Q Exactive Hybrid Quadrupole-Orbitrap Mass Spectrometer (Thermo Scientific). A home-made fused silica capillary column (75 μm × 150 mm, Upchurch, Oak Harbor, WA, USA) packed with C18 resin (300 Å, 5 μm, Varian Lexington, MA, USA) was used as the analytical column. Xcalibur 2.1.2 software was used with the Q Exactive mass spectrometer in data-dependent acquisition mode. Ten data-dependent MS/MS scans at 27% normalized collision energy (HCD) were performed, after which, a single full-scan mass spectrum in Orbitrap (400–1,800 m/z, 60, 000 resolution) was conducted.

### Statistical analysis

Osteoclast numbers in five random fields viewed under light microscopy were counted in both groups from Day 1 to Day 9. Furthermore, the mean size of mature osteoclasts and the total area of the resorbed pits were assessed with Adobe Photoshop CS3 and Image J software. Statistical differences between parametric data sets were assessed using Student’s *t*-test. A value of *P* < 0.05 was considered to indicate statistical significance.

LC-MS/MS data were analyzed using the Thermo Scientific Proteome Discoverer software suite 1.3 with the SEQUEST search engine and the mouse FASTA database from UniProt (released on July 9, 2014). At least 1 unique peptide per protein had to be identified to list the protein as a hit. In the SEQUEST search engine, full trypsin specificity was selected, two missed cleavages were allowed, carbamidomethylation (C) and TMT 6-plex (K and peptide N-terminal) were set as the static modification, oxidation (M) was set as the dynamic modification, precursor ion mass tolerances were set at 20 ppm for all MS data acquired using an Orbitrap mass analyzer, and the fragment ion mass tolerance was set as 20 mmu for all MS/MS spectra acquired. The ratio of proteins labeled with TMT^6^-127 and TMT^6^-126 was adjusted using the β-actin ratio value as the internal control. The thresholds for downregulation and upregulation were set at 0.6 and 1.7 respectively. In addition, the UniprotKB/Swiss-Prot accession numbers were converted into Entrez Gene ID for subsequent analysis using UniProt online ID Mapping. The PANTHER Classification System online was used to classify the proteins. The WebGestalt online toolkit was used to conduct enrichment analysis. Cytoscape 3.1.1 software was used to visualize the protein-protein interactions matching the online pathway database and the significance level was set at 0.0001. The mass spectrometry proteomics data have been deposited to the ProteomeXchange Consortium [32] via the PRIDE partner repository with the data identifier PXD001935.

## Additional file

Additional file 1:
**Details of differentially expressed proteins between normal and low serum model.**
